# Selective Inhibition of PKC*β*2 Restores Ischemic Postconditioning-Mediated Cardioprotection by Modulating Autophagy in Diabetic Rats

**DOI:** 10.1155/2020/2408240

**Published:** 2020-04-03

**Authors:** Yafeng Wang, Lu Zhou, Wating Su, Fengnan Huang, Yuan Zhang, Zhong-yuan Xia, Zhengyuan Xia, Shaoqing Lei

**Affiliations:** ^1^Department of Anaesthesiology, Renmin Hospital of Wuhan University, Wuhan, China; ^2^Department of Anesthesiology, The University of Hong Kong, Hong Kong SAR, China; ^3^Department of Anesthesiology, Affiliated Hospital of Guangdong Medical University, Zhanjiang 524001, China

## Abstract

Diabetic hearts are more susceptible to myocardial ischemia/reperfusion (I/R) injury and less sensitive to ischemic postconditioning (IPostC), but the underlying mechanisms remain unclear. PKC*β*2 is preferentially overactivated in diabetic myocardium, in which autophagy status is abnormal. This study determined whether hyperglycemia-induced PKC*β*2 activation resulted in autophagy abnormality and compromised IPostC cardioprotection in diabetes. We found that diabetic rats showed higher cardiac PKC*β*2 activation and lower autophagy than control at baseline. However, myocardial I/R further increased PKC*β*2 activation and promoted autophagy status in diabetic rats. IPostC significantly attenuated postischemic infarct size and CK-MB, accompanied with decreased PKC*β*2 activation and autophagy in control but not in diabetic rats. Pretreatment with CGP53353, a selective inhibitor of PKC*β*2, attenuated myocardial I/R-induced infarction and autophagy and restored IPostC-mediated cardioprotection in diabetes. Similarly, CGP53353 could restore hypoxic postconditioning (HPostC) protection against hypoxia reoxygenation- (HR-) induced injury evidenced by decreased LDH release and JC-1 monomeric cells and increased cell viability. These beneficial effects of CGP53353 were reversed by autophagy inducer rapamycin, but could be mimicked by autophagy inhibitor 3-MA. It is concluded that selective inhibition of PKC*β*2 could attenuate myocardial I/R injury and restore IPostC-mediated cardioprotection possibly through modulating autophagy in diabetes.

## 1. Introduction

Ischemic heart disease (IHD) is one of the most common perioperative complications with high mortality and disability particularly in patients with diabetes [1]. The most effective treatment for IHD is to restore the blood perfusion of ischemic myocardium, but paradoxically, this may cause lethal heart injury, termed “myocardial ischemia-reperfusion (I/R) injury “ [2]. Ischemic postconditioning (IPostC), achieved by transient brief interruptions of reperfusion by ischemic episodes, has been considered as an effective maneuver combat lethal reperfusion injury [3]. However, in diabetic condition, the hearts are more vulnerable to myocardial I/R injury and less or not responsive to IPostC [4], but the underlying mechanisms are still unclear.

Numerous studies suggest the activation of protein kinase C (PKC), a family of serine/threonine kinases with important physiological functions, is potentially responsible for the exacerbation of myocardial I/R injury in diabetes [5, 6]. However, the role of PKC in myocardial I/R injury is complicated by multiple isoforms, each with varying cellular distribution and opposing function at times [7–9]. Of the various isoforms of PKC, the activation of PKC*β*2 induced by hyperglycemia is most frequently implicated in cardiovascular complications in diabetes [10, 11]. Several studies have shown that PKC*β* activation negatively modulates mitochondrial energy status and autophagy [12, 13]. Autophagy is an important cellular self-protection mechanism that eliminates misfolded proteins and damaged organelles. However, autophagy just like a double-edged sword, excessive or low levels of autophagy, may result in harmful or damaging effects [14]. It has been shown that diabetes exhibits abnormal autophagy [15], and myocardial I/R injury exacerbates the dysfunctional autophagy activity [16, 17]. Our previous study has shown that selective inhibition of PKC*β*2 ameliorates myocardial I/R injury in diabetic rats [6]. Moreover, regulation of autophagy improves cardiac function [18, 19], ameliorates myocardial I/R injury, and restores IPostC cardioprotection in diabetes [20]. Furthermore, under the circumstance of LPS-induced oxidative stress and cellular injury in cultured cardiomyocytes, overactivation of PKC*β*2 was associated with autophagy activation [21]. However, the roles of PKC*β*2 in autophagy status and in particular the impact of their potential interaction on the loss of IPostC cardioprotection in diabetes have not been elucidated. In the present study, we hypothesize that hyperglycemia-induced PKC*β*2 activation involves autophagy abnormality. Our data suggest that the selective inhibition of PKC*β*2 attenuates myocardial I/R injury and restores IPostC cardioprotection by inhibiting autophagy in diabetes.

## 2. Materials and Methods

### 2.1. Experimental Animals and Induction of Type 1 Diabetes

This study was conformed to the regulations of Guide for the Care and Use of Laboratory Animals of the National Institutes of Health (NIH Publication No. 80–23) and approved by the Institutional Animal Care and Use Committee of Wuhan University. Male Sprague-Dawley rats (230 ± 10 g, 7-8weeks) were purchased from Beijing Vital River Laboratory Animal Technology Co. Ltd. All the rats were housed in the Animal Centre of Renmin Hospital of Wuhan University with a 12-hour (h) light-dark cycle and a standard environment. After 3 days of adaptive feeding, the rats were fasted for 12 hours and then administered single intraperitoneal injection of streptozotocin (STZ) (60 mg/kg, Sigma-Aldrich, St. Louis, MO, USA) dissolved in citrate buffer to induce diabetes as we described. [22] The control rats were administered single intraperitoneal injection of the same volume of citrate buffer. After 72 hours of the injection, random blood glucose was measured, and the glucose level >16.7 mmol/l were considered diabetic and used for the study.

### 2.2. Myocardial I/R Injury Models

The fourth intercostal space on the left side of the rat was opened to expose the heart. A white-lined blood vessel, which is the LAD (left anterior coronary artery), is seen at the lower edge of the left atrial appendage. The 6-0 band surgical needle is used for thread ligation. When the ventricle turns white and the electrocardiogram shows that the ST segment immediately elevated and the T wave becomes high, these suggest myocardial ischemia. The I/R injury model was achieved by 30 min ischemia and followed by 120 min reperfusion. The sham rats underwent the same surgical procedures without ligation. IPostC was induced by the cycles of 10 s of reperfusion and ischemia after ischemia for 30 min as we described previously [23].

### 2.3. Experimental Protocols and Drug Treatment

After 8 weeks of STZ injection, the diabetic (DM) and age-matched control (Ctrl) rats were randomly divided into six groups (*n* = 8): (1) Ctrl+sham (S); (2) Ctrl+I/R; (3) Ctrl+IPostC; (4) DM+S; (5) DM+I/R; and (6) DM+IPostC. Furthermore, to evaluate the role of the PKC*β*2 in IPostC-induced cardioprotection, we conducted further experiments in the following groups (*n* = 8): (1) DM+I/R+CGP53353 and (2) DM+IPostC+CGP53353. CGP53353 (Sigma-Aldrich, USA) is a selective inhibitor of PKC*β*2 (IC50 values are 0.41 *μ*mol/l for PKC*β*2 and 3.8 *μ*mol/l for PKC*β*1) [24], which was dissolved in DMSO as stocking inhibitor, and then diluted with normal saline for rats or DMEM for cell culture (dilution is often more than 1 : 10000) as working inhibitor before injection. CGP53353 (10 *μ*g/kg) was intravenous injection pumped 10 minutes before ligation of the LAD.

### 2.4. Determination of Myocardial Infarct Size

At the end of 120 min of reperfusion, myocardial infarct size was measured by staining with 0.25% Evans Blue dye (Sigma–Aldrich) and 1% 2,3,5-triphenyltetrazolium chloride (TTC, Sigma–Aldrich) as we described previously [25]. The area unstained by Evans Blue dye was identified as the area at risk (AAR), and the area unstained by TTC was considered as the infarcted tissue. Myocardial infarct size was showed as a percentage of the AAR (% of AAR).

### 2.5. Measurement of Creatine Kinase-MB (CK-MB) and Lactate Dehydrogenase (LDH)

The levels of serum CK-MB are specific indicators reflecting the extent of myocardial damage. After 120 min of reperfusion, the arterial blood was collected, and then the serum CK-MB was measured by using a commercial ELISA assay kit according to the manufacturer's instructions (Jiancheng, Nanjing, China).

### 2.6. Cell Culture

Embryonic rat cardiomyocyte-derived H9C2 cell line was obtained from the Cell Bank of the Chinese Academy of Sciences (Shanghai, China). Cells were cultured in DMEM (Gibco Laboratories) containing 10% (*v*/*v*) FBS (Gibco Laboratories) and 1% antibiotics under a humidified atmosphere of 5% CO_2_ and 95% air at 37°C as we previously reported. [21] The cells were randomly divided into seven groups: (1) High glucose (30 mM, HG); (2) HG+HR; (3) HG+HR+CGP53353 (1 *μ*M); (4) HG+HPostC; (5) HG+HPostC+CGP53353 (1 *μ*M); (6) HG+HPostC+3-MA (10 mM); and (7) HG+HPostC+CGP53353+rapamycin (100 nM). HG was induced by 50% glucose injection at a final concentration of 30 mM for 48 h. All treatments were administered 1 h before HR. After HG exposure for 48 h, the HR procedure was performed. For HR exposure, cells were maintained under anoxic conditions in chambers gassed with a mixture of 94% N2, 5% CO2, and 1% O2 at 37°C for 4 h and reoxygenation for 2 h. HPostC was achieved after 4 h of hypoxia and before 2 h of reoxygenation, including 3 cycles of 5 min of reoxygenation and hypoxia.

### 2.7. Determination of Mitochondrial Membrane Potential (MMP)

MMP was measured by JC-1 staining (Beyotime, China) according to the experimental protocol. Firstly, H9C2 cells were cultured in a 6-well plate with corresponding treatment. Then, removed corresponding medium and wash 3 times with PBS. After that, cells were incubated with 1 ml of 10 *μ*g/ml JC-1 dye for 25 min at 37°C. Cells were subsequently washed three times with JC-1 buffer. Finally, cells were scanned with epifluorescence. Under a high level of MMP, JC-1 aggregates into the matrix of the mitochondria to form a polymer (J-aggregates), which can produce red fluorescence; on the contrary, JC-1 cannot aggregate mitochondria in the matrix, JC-1 is a monomer at this time, and green fluorescence can be produced.

### 2.8. Western Blot

Heart tissue and cultured cells were homogenized in RIPA buffer containing the phosphatase inhibitor. The homogenates was centrifuged to collect the supernatant as total protein preparations, and the protein concentration of each sample was measured using a BCA kit (Beyotime). Equal amounts of proteins were separated via SDS-PAGE and transferred to PVDF membranes for immunoblot analysis as described previously [21]. Primary antibodies against GAPDH (1 : 1000 dilution, Cell Signaling Technology, USA), phospho-PKC*β*2 (Ser^660^) (1 : 1000 dilution, Cell Signaling Technology, USA), Beclin-1 (1 : 1000 dilution, Abcam company, USA), P62 (1 : 1000 dilution, Abcam company, USA), and LC3 (1 : 1000 dilution, Cell Signaling Technology, USA) were used in the present study. GAPDH was used as a loading control and all the results were presented as percent change relative to control measurement.

### 2.9. Statistical Analysis

Data are expressed as mean ± SD. All statistical analyses were performed by one-way or two-way analysis of variance (ANOVA) using an SPSS 24.0 Software. A value of *P* < 0.05 was considered to statistically significant difference.

## 3. Results

### 3.1. IPostC-Reduced Myocardial I/R Injury in Control but Not in Diabetic Rats

**Figure 1:**
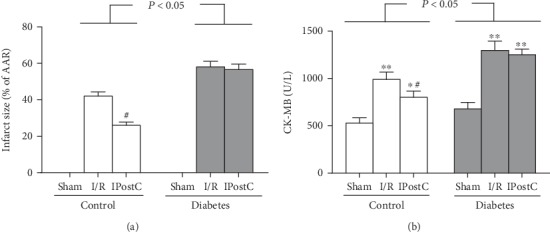
Ischemic postconditioning (IPostC) reduced myocardial ischemia/reperfusion (I/R) injury in control but not in diabetic rats. (a) Infract size and (b) CK-MB. All results are expressed as mean ± SD, *n* = 8. ^∗^*P* < 0.05, ^∗∗^*P* < 0.01 vs. the corresponding sham group, ^#^*P* < 0.05 vs. the corresponding I/R group.

In the present study, we first investigated the effects of IPostC on myocardial I/R injury in STZ-induced diabetic rats and age-matched control rats. After STZ induction for 8 weeks, the diabetic rats showed higher water intake, food consumption, and plasma glucose, accompanied with lower body weight than control rats (Table 1). When the rats underwent myocardial I/R, the infarct size (% AAR) and plasma CK-MB in diabetic rats were larger than that in the corresponding control rats (Figures 1(a) and 1(b)). IPostC significantly reduced the infarct size and CK-MB in age-matched control rats, but failed to elicit protective effects in diabetic rats (Figures 1(a) and 1(b)), indicating that IPostC-mediated cardioprotection was compromised by diabetes.

### 3.2. IPostC-Reduced Myocardial I/R-Induced PKC*β*2 Activation and Autophagy in Control but Not in Diabetic Rats

**Figure 2:**
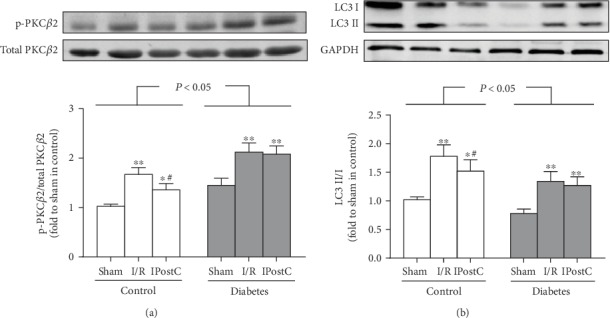
Ischemic postconditioning (IPostC) reduced myocardial ischemia/reperfusion- (I/R-) induced PKC*β*2 activation and autophagy in control but not in diabetic rats. (a) Representative Western blot of p-PKC*β*2 (ser^660^) compared with total PKC*β*2 and (b) representative Western blot of LC3 II/I compared with GAPDH. All results are expressed as mean ± SD, *n* = 8. ^∗^*P* < 0.05, ^∗∗^*P* < 0.01 vs. the corresponding sham group; ^#^*P* < 0.05 vs. the corresponding ischemia/reperfusion (I/R) group.

We previously demonstrated that hyperglycemia-induced PKC*β*2 activation [6] and downregulated autophagy [20] were involved in the decreased tolerance to myocardial I/R injury in diabetes. In the present study, we determined whether IPostC could affect PKC*β*2 activation and autophagy status in diabetic rats and age-matched control rats. As shown in Figure 2(a), diabetes significantly increased PKC*β*2 phosphorylation on Ser^660^ without influencing total PKC*β*2 expression (indicating PKC*β*2 activation), accompanied with downregulated autophagy status detected by decreased ratio of LC-3II/LC-3I (Figure 2(b)). Myocardial I/R significantly increased PKC*β*2 phosphorylation and the ratio of LC-3II/LC-3I as compared with the corresponding sham group both in control and diabetic rats, and these alterations induced by myocardial I/R were attenuated by IPostC in control but not in diabetic rats (Figure 2).

### 3.3. Selective Inhibition of PKC*β*2 with CGP53353 Restores IPostC-Mediated Cardioprotection in Diabetic Rats

**Figure 3:**
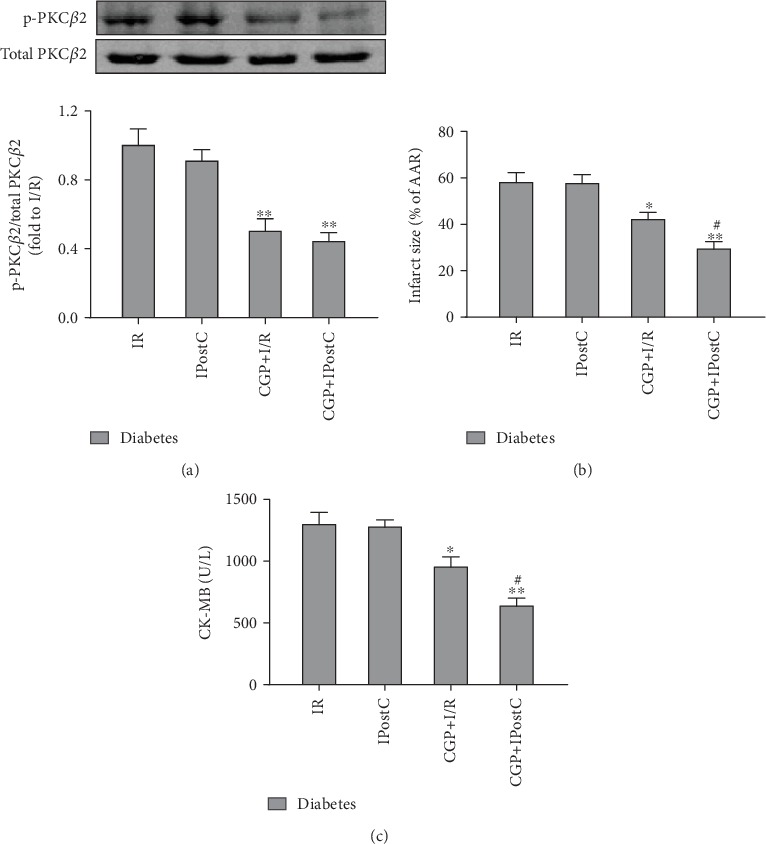
Selective inhibition of PKC*β*2 with CGP53353 (CGP) restores IPostC-mediated cardioprotection in diabetic rats. (a) Representative Western blot of p-PKC*β*2 (ser^660^) compared with total PKC*β*2, (b) infract size, and (c) CK-MB. All results are expressed as mean ± SD, *n* = 8. ^∗^*P* < 0.05, ^∗∗^*P* < 0.01 vs. I/R group; ^#^*P* < 0.05 vs. CGP+I/R group.

Based on the above findings, we next investigated the treatment effects of CGP53353 (a selective inhibitor of PKC*β*2) on IPostC in diabetic rats. The CGP53353 vehicle had no effects on infarct size and CK-MB (data not shown). As shown in Figure 3(a), CGP53353 could well inhibit PKC*β*2 phosphorylation both in I/R and IPostC group, though there was no significant difference in PKC*β*2 phosphorylation between them. As shown in Figures 3(b) and 3(c), IPostC had no significant effects on postischemic infarct size and plasma CK-MB in diabetic rats. However, with the treatment with CGP53353, both infarct size and CK-MB in diabetic rats were significantly reduced in the I/R group, which were further decreased by IPostC. These suggest that CGP53353 treatment could restore IPostC-mediated cardioprotection in diabetic rats and confer synergistic or added cardioprotection.

### 3.4. Effects of CGP53353 on Myocardial Autophagy Status in Diabetic Rats

**Figure 4:**
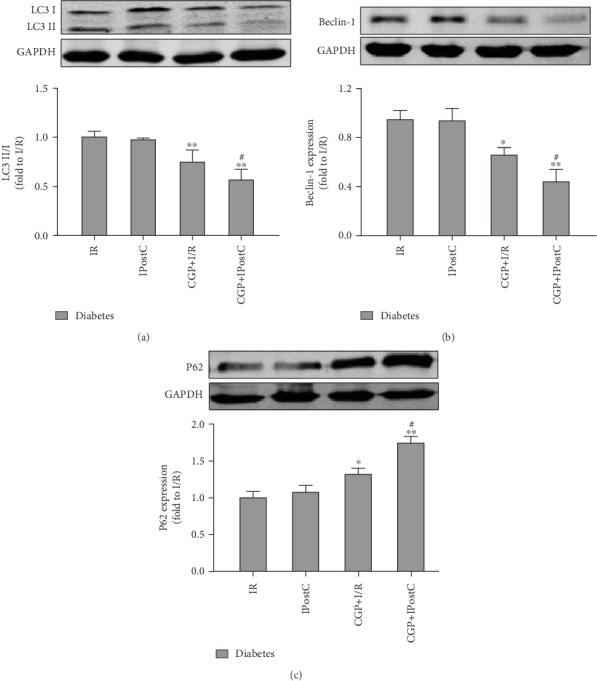
Effects of CGP53353 (CGP) on autophagy-associated proteins in diabetes rats subjected to myocardial ischemia/reperfusion (I/R) and ischemic postconditioning (IPostC). (a) Representative Western blot of LC3 II/I with GAPDH as loading control, (b) representative Western blot of Beclin-1 with GAPDH as loading control, and (c) representative Western blot of P62 with GAPDH as loading control. All results are expressed as mean ± SD, *n* = 8. ^∗^*P* < 0.05, ^∗∗^*P* < 0.01 vs. I/R group; ^#^*P* < 0.05 vs. CGP+I/R group.

We then evaluated the treatment effects of CGP53353 on autophagy status in diabetic rats subjected to myocardial I/R and IPostC. Myocardial I/R significantly increased the ratio of LC3II/LC3I (Figure 4(a)) and Beclin-1 expression (Figure 4(b)), accompanied with decreased P62 expression (Figure 4(c)). IPostC alone did not affect these alterations. By contrast, all these alterations induced by myocardial I/R were significantly attenuated by PKC*β*2 inhibition with CGP53353, which were further attenuated by combination of CGP53353 and IPostC (Figure 4).

### 3.5. Selective Inhibition of PKC*β*2 with CGP53353 Restored HPostC Protection against HR-Induced Cell Injury

**Figure 5:**
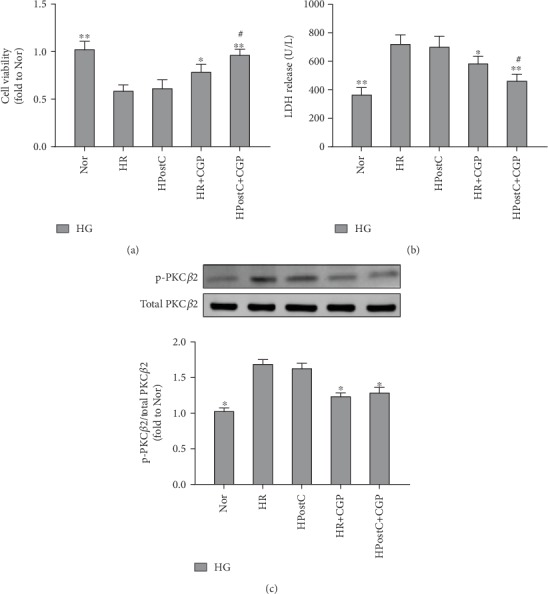
Selective inhibition of PKC*β*2 with CGP53353 (CGP) restored hypoxic postconditioning (HPostC) protection against hypoxia reoxygenation- (HR-) induced cell injury. (a) Cell viability, (b) LDH release, and (c) representative Western blot of p-PKC*β*2 (ser^660^) compared with total PKC*β*2. All results are expressed as mean ± SD, *n* = 8. ^∗^*P* < 0.05, ^∗∗^*P* < 0.01 vs. HR group; ^#^*P* < 0.05 vs. HR+CGP group.

We confirmed the treatment effects of CGP53353 on HPostC in H9C2 cells during HG conditions. As shown in Figures 5(a) and 5(b), HR stimulation significantly decreased cell viability and increased LDH release as compared with the normoxic group, accompanied with increased activation of PKC*β*2 (Figure 5(c)), and these alterations were not affected by HPostC. Selective inhibition of PKC*β*2 with CGP53353 treatment significantly attenuated HR-induced decrease of cell viability and increase of LDH release, which were further attenuated by the combination of HPostC (Figures 5(a) and 5(b)).

### 3.6. Role of Autophagy in CGP53353-Restored HPostC Protection in H9C2 Cells

**Figure 6:**
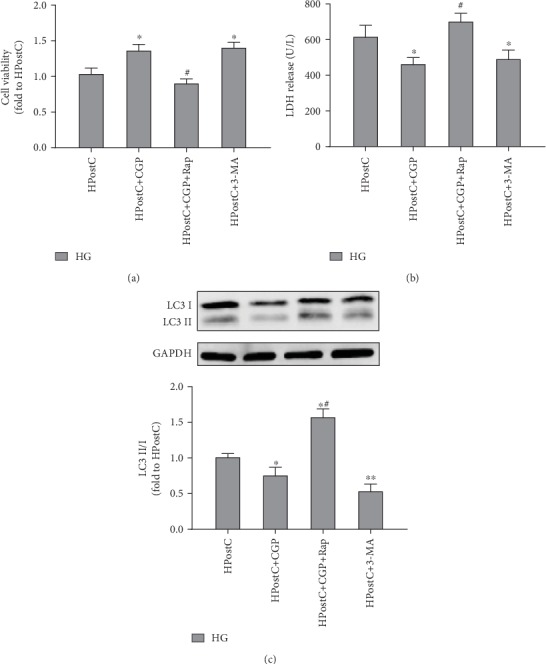
Effects of CGP53353 (CGP), 3-methylrapadenine (3-MA), and rapamycin (Rap) on cell viability. (a) Cell viability, (b) LDH release, and (c) the expression of LC3II/I in H9C2 cardiomyocytes exposed to hypoxic postconditioning (HPostC) in the condition of high glucose (HG). All results are expressed as mean ± SD, *n* = 8. ^∗^*P* < 0.05 vs. HPostC group; ^#^*P* < 0.05 vs. HPostC+CGP group.

To further investigate the role of autophagy in the beneficial effects of CGP53353, we also used autophagy inducer rapamycin and inhibitor 3-MA to treat the cells. As shown in Figures 6(a) and 6(b), 3-MA had similar effects to CGP53353 in restoring HPostC protection from cell injury detected by increased cell viability and decreased LDH release. However, after the addition of autophagy inducer Rap, the beneficial effects of CGP53353 were abolished (Figures 6(a) and 6(b)). We then determined the treatment effects of CGP53353 on LC3 II and LC3 I expression. As shown in Figure 6(c), CGP significantly attenuated the increase of the ratio of LC3II/LC3I induced by HR, which was further attenuated by the addition of HPostC. Similar effects were shown by the treatment of autophagy inhibitor 3-MA. Autophagy inducer Rap abolished the effects of CGP53353 and HPostC on the ratio of LC3II/I induced by HR.

### 3.7. Effects of CGP on Mitochondrial Membrane Potential (MMP) in H9C2 Cells Exposed to HR and HPostC

**Figure 7:**
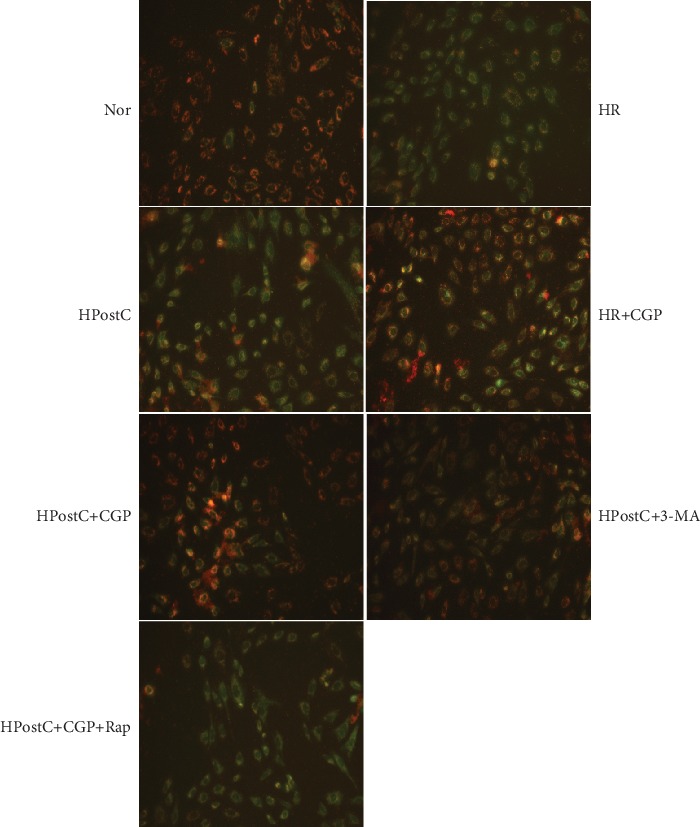
Effects of CGP53353 (CGP), 3-methylrapadenine (3-MA), and rapamycin (Rap) on the loss of mitochondrial membrane potential (MMP) by detecting JC-1 monomeric cells in cultured H9C2 cardiomyocytes exposed to hypoxia reoxygenation (HR) or hypoxic postconditioning (HPostC) in high glucose condition.

We also determined the loss of MPP by detecting JC-1 monomeric cells to evaluate mitochondrial damage in H9C2 cells exposed to HG and HR. As shown in Figure 7, HR insult significantly increased the percentage of JC-1 monomeric cells, which was not affected by HPostC alone. CGP53353 treatment significantly reduced the percentage of JC-1 monomeric cells induced by HR, which was further reduced by the combined use of HPostC. Similar effects were shown by the treatment of autophagy inhibitor 3-MA. In contrast, autophagy inducer Rap abolished the effects of CGP and HPostC on MPP.

## 4. Discussion

In the present study, we have demonstrated that the cardioprotection of IPostC is compromised in diabetes, which is associated with the excessive activation of PKC*β*2 induced by hyperglycemia. Selective inhibition of PKC*β*2 restored IPostC cardioprotection by modulating autophagy status. To our knowledge, this is the first study to investigate the relative roles of PKC*β*2 and autophagy in IPostC in diabetes.

IPostC is well demonstrated to be effective against myocardial I/R injury in nondiabetic conditions [26, 27]. IPostC is achieved by transient brief interruptions of reperfusion by ischemic episodes, so it can be used for treatment of unpredictable myocardial ischemia and confer greater clinical application prospects, such as cardiac interventional surgery [28–30]. However, the presence of diabetes or hyperglycemia renders the hearts more resistant to the infarct size-limiting effects of IPostC [31–33]. In the present study, we found that IPostC significantly reduced myocardial I/R injury in age-matched control rats but not in diabetic rats. Similar effects of HPostC on HR injury were found in H9C2 cells. These results indicate that diabetes may blunt IPostC cardioprotection.

It is well demonstrated that the activation of PKC*β* plays a critical role in myocardial I/R injury in nondiabetic rodents [34]. In diabetic condition, PKC*β* is excessively activated by hyperglycemia in vascular complications of diabetes [35]. We further found that PKC*β*2, but not PKC*β*1, was preferentially overactivated in the myocardium [10], which resulted in the increased vulnerability of myocardial I/R injury in diabetes [6]. Our results showed that diabetes significantly increased myocardial PKC*β*2 activation, which were further increased by myocardial I/R insult. We speculated that the PKC*β*2 activation was also attributable to the loss of IPostC cardioprotection in diabetes. Indeed, after the treatment of CGP53353, a selective inhibitor of PKC*β*2, myocardial I/R-induced postischemic infarct size, and CK-MB were significantly attenuated, which were further attenuated by IPostC. Similarly, HPostC significantly reduced HG and HR-induced LDH release and JC-1 monomeric cells and increased cell viability in the presence of CGP53353. Thus, the loss of IPostC cardioprotection in diabetes may be in part explained by PKC*β*2 activation, and selective inhibition of PKC*β*2 may be a useful therapy to preserve IPostC cardioprotection.

Autophagy occurs at basal level and is critical for maintaining the homeostasis of cells through removing damaged organelles and misfolded proteins [36]. It is believed that autophagy plays a “double-edged sword” role in the development of cardiovascular diseases, and excessive or low levels of autophagy may lead to a negative impact [14]. Diabetes exhibits abnormal autophagy [15], and myocardial I/R injury exacerbates the dysfunctional autophagy activity [16, 17]. During autophagy, misfolded protein and damaged organelles are captured in double-membraned vesicles (autophagosomes) and degraded through lysosomal fusion [37]. P62 delivers ubiquitinated cargoes for autophagic degradation, and activating autophagy can reduce the expression of P62 [38]. Additionally, Beclin-1 and the ratio of LC3II/LC3I are proved to be autophagosomal markers in mammals [39]. The current study confirmed the impaired autophagy function in STZ-induced type-1 diabetes, and myocardial I/R excessively induced autophagy status indicated by increased ratio of LC3II/LC3I and Beclin-1 expression with decreased p62. It has been shown that PKC*β* negatively modulates mitochondrial energy status and autophagy, and inhibition of PKC*β* with pharmacological inhibitor shows an increase in autophagy both in vitro and in vivo study [12, 13]. In the present study, we found selective inhibition of PKC*β*2 with CGP53353 attenuated autophagy dysfunction induced by diabetes and myocardial I/R and restored IPostC cardioprotection in diabetic rats. Similar effects of CGP53353 on HPostC were mimicked by modulating autophagy with the treatment of autophagy inhibitor 3-MA in H9C2 cells exposed to HG and HPostC, but the beneficial effects of CGP53353 were reversed by autophagy inducer rapamycin. Our results indicate that selective inhibition of PKC*β*2 activation may modulate autophagy to a moderate level to exert beneficial effects.

In summary, the results of the current study demonstrate that the compromised cardioprotection of IPostC is associated with excessive activation PKC*β*2 activation, which contributes to autophagy dysfunction. Selective inhibition of PKC*β*2 restores IPostC cardioprotection possibly through modulating autophagy status. Therefore, PKC*β*2 blockade may be a useful approach for attenuating myocardial I/R injury and preserving the effectiveness of IPostC.

## Figures and Tables

**Table 1:** General characteristics of diabetic rats at the end of the study.

	Control	Diabetes
Body weight (g)	497.67 ± 18.80	308.00±16.04^∗∗^
Water intake (ml/kg/day)	131.17 ± 18.89	722.83±42.90^∗∗^
Food consumption (g/kg/day)	75.17 ± 4.07	208.00±15.81^∗∗^
Blood glucose (mmol/l)	5.38 ± 0.62	29.68±3.06^∗∗^

All the results are expressed as mean ± SD, *n* = 8. Differences in general characteristics were determined by one-way analysis of variance (ANOVA) followed by Tukey's test. ^∗∗^*P* < 0 01 vs. the control group.

## Data Availability

The data used to support the findings of this study are included within the article.
